# Outcomes Beyond 10 Years After Transcatheter Aortic Valve Implantation in High‐Risk Patients With Severe Aortic Valve Stenosis

**DOI:** 10.1002/ccd.31677

**Published:** 2025-06-12

**Authors:** Ellen Dietze, Ines Richter, Oliver Dumpies, Johannes Rotta detto Loria, Nicolas Majunke, Hans‐Josef Feistritzer, Philipp Kiefer, David Holzhey, Thilo Noack, Axel Linke, Norman Mangner, Steffen Desch, Michael Borger, Holger Thiele, Mohamed Abdel‐Wahab

**Affiliations:** ^1^ Department of Cardiology Heart Center Leipzig at Leipzig University Leipzig Germany; ^2^ Department of Cardiac Surgery Heart Center Leipzig at Leipzig University Leipzig Germany; ^3^ Department for Internal Medicine and Cardiology, Heart Centre Dresden, Faculty of Medicine and University Hospital Carl Gustav Carus TUD Dresden University of Technology Dresden Germany

## Abstract

**Background:**

Limited data is available on long‐term outcomes and valve durability measures of transcatheter aortic valve implantation (TAVI).

**Aims:**

This study sought to assess clinical and echocardiographic outcomes of high‐risk patients during the early experience of TAVI with a follow‐up period extending beyond 10 years.

**Methods:**

Patients were included who had undergone TAVI at the Heart Center Leipzig between 2006 and 2012. Valve durability measures including moderate and severe structural valve deterioration (SVD) were defined according to the updated standardized definitions of the Valve Academic Research Consortium 3 (VARC‐3).

**Results:**

A total of 1825 patients were included. The 12‐year mortality rate was 92.8%. At 12 years post‐TAVI, the cumulative incidence of SVD was 9.8% in the total population. Complete echocardiographic follow‐up at ≥ 9 years was available for 56 patients. The median follow‐up of this subgroup was 10.0 years. Moderate SVD was observed in three patients of the echocardiographic follow‐up cohort (5.4%) and severe SVD in two patients (3.6%). Self‐expanding (SE) transcatheter heart valves (THV) had a significantly lower rate of any SVD (2.8% vs 20.0%, *p* = 0.030) than balloon‐expandable (BE) THV.

**Conclusion:**

Long‐term mortality rates in high‐risk patients treated with TAVI more than 10 years ago are substantial, which limits assessment of valve durability measures. In a subgroup of survivors beyond 9 years, the cumulative incidence of SVD was low.

**Abbreviations**
AR: aortic regurgitation
AS: aortic stenosis
BE: balloon‐expandable
BVF: bioprosthetic valve failure
CI: confidence interval
CT: computed tomography
DVI: doppler velocity index
EOA: effective orifice area
HR: hazard ratio
LVEF: left ventricular ejection fraction
NYHA: New York Heart Association
PARTNER: placement of aortic transcatheter valves
Pmax: peak transvalvular pressure gradient
Pmean: mean transvalvular pressure gradient
SAVR: surgical aortic valve replacement
SE: self‐expanding
STS: society of thoracic surgeons
SVD: structural valve deterioration
TAVI: transcatheter aortic valve implantation
THV: transcatheter heart valve
TTE: transthoracic echocardiography
VARC: Valve Academic Research Consortium
ViV: valve‐in‐valve

## 1 Introduction

Aortic stenosis (AS) is a common valvular heart disease with a prevalence of up to 10.0% in the eighth decade of life [1]. Once AS becomes symptomatic, rapid intervention is recommended [2]. Transcatheter aortic valve implantation (TAVI) is nowadays universally accepted as the treatment of choice for elderly patients with severe AS [3]. One reason for setting age thresholds for TAVI remains the limited knowledge of long‐term durability of transcatheter heart valves (THV).

As surgical aortic valve replacement (SAVR) has been performed since 1960 [4], several studies have evaluated the long‐term performance of surgical bioprosthetic valves and reported structural valve deterioration (SVD) rates ranging from 7.0% to 30.0% 10 years after surgery, depending on the definition applied [5]. Within the last years, TAVI technology has constantly improved, and the procedure is being utilized with a rapidly increasing frequency since its first introduction in 2002 [4]. Randomized trials and meta‐analyses demonstrated the noninferiority of TAVI compared to SAVR in terms of mortality and valve function over time [6, 7, 8]. Furthermore, a few studies reported lower rates of SVD with TAVI compared to SAVR, with a similar rate of bioprosthetic valve failure (BVF) at 8–10 years [9, 10, 11]. In studies reporting outcomes of TAVI up to 10 years, the cumulative incidence of any SVD ranged between 4.7% and 6.5% [12, 13]. However, only limited data exists on long‐term outcomes beyond 10 years. Therefore, the objective of the present study is to evaluate long‐term mortality and valve durability after TAVI with a follow‐up period of up to 12 years, and to provide insights from a historical high‐risk cohort of patients treated at a single, high‐volume center in Germany.

## 2 Methods

### 2.1 Study Population and Patient Selection

**Figure 1:**
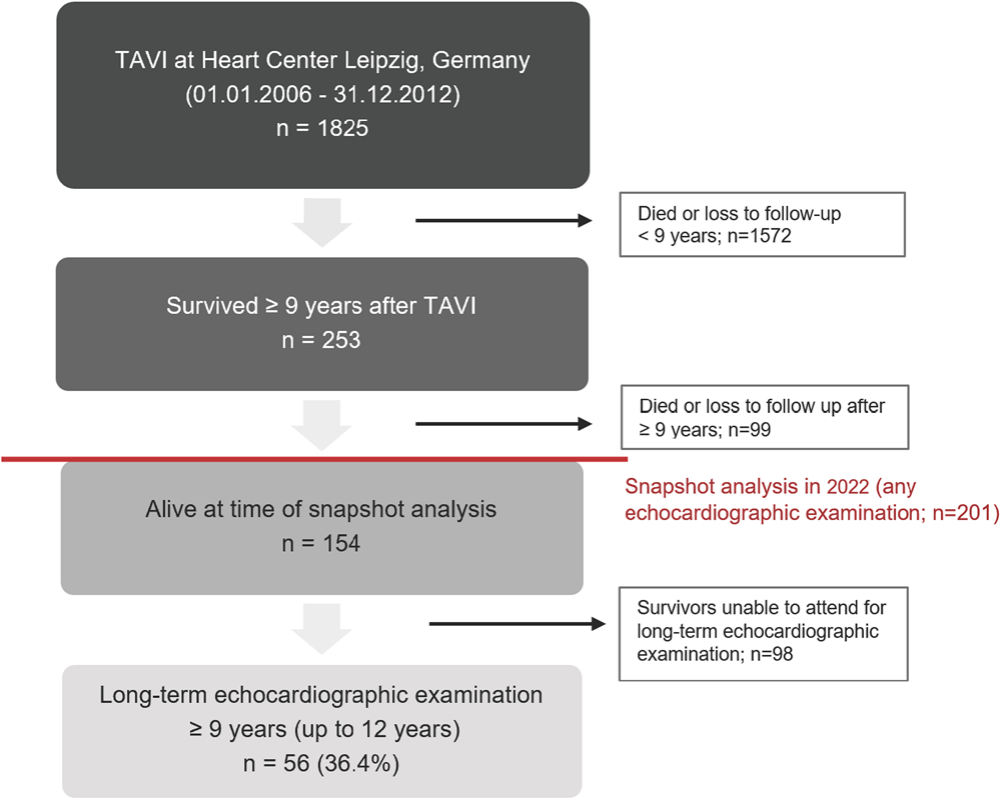
Study flow chart. An illustration of the transition from the initial study population to the follow‐up subgroup. Data were censored for patients with loss of follow‐up. TAVI = transcatheter aortic valve implantation. [Color figure can be viewed at wileyonlinelibrary.com]

The study cohort included patients who underwent an elective or urgent TAVI procedure at the Heart Center Leipzig at Leipzig University in Germany between 2006 and 2012. The final decision whether to perform TAVI or SAVR was made by a multidisciplinary Heart Team for each individual case. The decision was mainly based on operative risk assessment, which was determined by the expected operative mortality (EuroSCORE, Society of Thoracic Surgery [STS] risk score) as well as factors such as age, frailty and pre‐existing medical conditions. Routine follow‐up visits were scheduled at 1‐, 3‐, 5‐, 7, and 10 years. To evaluate mortality rates and THV durability for patients with a follow‐up of ≥ 9 years, we performed a snapshot analysis in 2022 and reanalyzed all available data. Survival data from the local institutional database, general practitioners or the patient's relatives were collected. Patients who were still alive were contacted and invited to an on‐site visit with clinical examination and transthoracic echocardiography (TTE). If an on‐site visit was not possible, echocardiographic data from hospital records or patients' local cardiologists were included. Patients who survived and underwent a full echocardiographic follow‐up at ≥ 9 years constituted a subpopulation for durability analysis, which was partially analyzed separately (Figure 1). Written informed consent was provided by all patients. The study population is part of the observational Leipzig TAVI Registry, which was approved by the responsible ethics committee. The study complies with the Declaration of Helsinki.

### 2.2 Procedural Details

All patients evaluated for TAVI underwent preprocedural diagnostic assessment and echocardiographic evaluation. Details of the procedure have been described previously [14, 15, 16, 17]. Both transfemoral and transapical TAVI cases were included. The available THVs for implantation included the self‐expanding (SE) CoreValve model (Medtronic, Minneapolis, MN, USA), the balloon‐expandable (BE) SAPIEN/SAPIEN XT prosthesis (Edwards Lifesciences, Irvine, CA, USA) and other less common SE valves. The implantation was performed in a hybrid operating theater by an interdisciplinary heart team.

### 2.3 Outcomes and Study Endpoints

The main objective of this study was to evaluate clinical and echocardiographic long‐term outcomes after TAVI as well as THV durability. In addition, we sought to identify specific factors associated with mortality and those that may affect early degeneration of THVs. Mortality rates were estimated at 1‐, 3‐, 5‐, 7‐, 9‐, 10‐, 11‐, and 12 years.

To assess THV durability, SVD was analyzed according to the criteria established by the Valve Academic Research Consortium (VARC‐3) [18]. SVD was defined as intrinsic changes of the valve leaflets, such as calcification, disruption, flail leaflet and/or leaflet fibrosis, and was differentiated into three degrees of severity. Stage 1 refers to morphological valve dysfunction without evidence of hemodynamic changes. Stage 2 describes moderate hemodynamic valve deterioration, defined as an increase of the mean transvalvular pressure gradient (Pmean) ≥ 10.0 mmHg compared to baseline leading to a Pmean of ≥ 20.0 mmHg, with an additional decrease in the aortic effective orifice area (EOA) ≥ 0.3 cm^2^ or ≥ 25.0%, a decrease in Doppler velocity index (DVI) ≥ 0.1 or ≥ 20.0% or an increase of ≥ 1 degree of intraprosthetic aortic regurgitation (AR) resulting in ≥ moderate AR. Severe SVD corresponding to stage 3 is described as an increase of the Pmean ≥ 20.0 mmHg resulting in a Pmean ≥ 30.0 mmHg in addition to a decrease in EOA ≥ 0.6 cm^2^ or ≥ 50.0%, a reduction in DVI of ≥ 0.2 or ≥ 40.0% or the increase of 2 grades resulting in severe intraprosthetic AR.

### 2.4 Statistical Analysis

Baseline characteristics are described as means with standard deviation or median with interquartile range, as appropriate. For the comparison of continuous variables between two groups, we used the unpaired *t* test or the Welch‐test/ANOVA‐test for normally distributed data or otherwise the Mann–Whitney‐*U*‐test. Paired continuous data were compared with the paired *t* test in case of normal distribution or otherwise with the Wilcoxon‐signed‐rank‐test. For the comparison of categorial variables we applied the *χ* ^2^ test or the Fisher's exact test, as appropriate.

Time to event analyses were performed using Kaplan–Meier analysis. For group comparisons, the Log‐Rank test was applied. To analyze parameters affecting mortality, a Cox‐regression analysis was performed using univariable and multivariable hazard models. To differentiate the influence of factors on short‐term versus long‐term mortality, a landmark analysis at 1‐year was also conducted. A competing risk analysis was performed to determine the cumulative incidence of SVD adjusted for mortality, and the Gray‐Test was used for comparisons. A two‐sided *p* < 0.05 was considered statistically significant. Statistical analysis was performed using SPSS, Version 28.0.1.1 (IBM Corp., Armonk, NY, USA) and R, Version 4.2.2 (r‐project, open source).

## 3 Results

### 3.1 Baseline Characteristics and Procedural Outcomes

A total of 1825 patients treated with TAVI between 2006 and 2012 were included. The median age of the patient population was 81.0 [77.0; 85.0] years and 59.2% were female. Most patients were at prohibitive or high risk for surgery with a median STS‐score of 7.5 [4.8; 11.9]. Baseline characteristics are shown in Table 1.

**Table 1:** Baseline clinical and echocardiographic characteristics of the total study population (*n* = 1825).

Clinical parameters at baseline	
Age, years	81.0 [77.0; 85.0]
Female	1080 (59.2%)
STS score	7.5 [4.8; 11.9]
Hypertension	1727 (94.6%)
Diabetes mellitus	836 (45.8%)
Coronary artery disease	947 (51.9%)
Carotid stenosis	326 (17.9%)
Peripheral arterial disease	245 (13.4%)
Previous stroke	184 (10.1%)
Previous myocardial infarction	247 (13.5%)
Pulmonary hypertension	614 (33.6%)
Chronic renal dialysis	64 (3.5%)
NYHA class III/IV	1468 (80.4%)
Preprocedural echocardiographic parameters	
LVEF, %	58.0 [46.1; 65.0]
EOA, cm^2^	0.6 [0.5; 0.8]
Pmax, mmHg	70.7 [55.9; 87.0]
Pmean, mmHg	44.0 [34.0; 55.2]
≥ moderate AR	319 (18.4%)

*Note:* Values are *n* (%), mean ± standard deviation or median [interquartile range].

Abbreviations: AR, aortic regurgitation; EOA, effective orifice area; LVEF, left ventricular ejection fraction; NYHA, New York Heart Association; Pmax, peak transvalvular pressure gradient; Pmean, mean transvalvular pressure gradient; STS, Society of Thoracic Surgeons

Indication for TAVI was AS in 94.5%, AR in 0.2%, and a combined valve disease in 2.0%. Only 3.3% of cases were performed as valve‐in‐valve (ViV) interventions for either degenerated surgical valves (95.0%) or THVs (5.0%). The intervention was performed through the transfemoral approach in 62.5% and through the transapical approach in 37.5%. Patients received SE CoreValve in 879 (48.2%) cases and BE SAPIEN/SAPIEN XT valve in 752 (41.2%). The remaining limited number of patients were treated with other SE THVs. Procedural details and echocardiographic outcomes are summarized in Table 2.

**Table 2:** Procedural details and echocardiographic outcomes of the total study population (*n* = 1825).

Approach	
Transfemoral aortic valve implantation	1140 (62.5%)
Transapical aortic valve implantation	685 (37.5%)
Indication for TAVI	
Aortic stenosis	1725 (94.5%)
Aortic regurgitation	3 (0.2%)
Combined aortic stenosis and aortic regurgitation	37 (2.0%)
Valve‐in‐valve intervention	60 (3.3%)
THV device	
Medtronic CoreValve	879 (48.2%)
Edwards SAPIEN/SAPIEN XT	752 (41.2%)
Other valves	194 (10.6%)
Symetis	74 (4.1%)
Portico	19 (1.0%)
Medtronic engager	48 (2.6%)
JenaValve	30 (1.6%)
Ventor	23 (1.3%)
Echocardiographic outcomes	
LVEF, %	60.0 [50.0; 65.0]
EOA, cm^2^	1.7 [1.4; 2.1]
Pmean, mmHg	9.0 [6.0; 12.0]
Pmax, mmHg	16.0 [12.0; 22.0]
≥ moderate AR	111 (6.8%)

*Note:* Values are *n* (%), mean ± standard deviation, or median [interquartile range].

Abbreviations: AR, aortic regurgitation; EOA, effective orifice area; LVEF, left ventricular ejection fraction; Pmax, peak transvalvular pressure gradient; Pmean, mean transvalvular pressure gradient; TAVI, transcatheter aortic valve implantation; THV, transcatheter heart valve.

Overall, 253 patients survived ≥ 9 years, of whom 154 patients were still alive at the time of the snapshot analysis. Among these, a full echocardiographic follow‐up could be obtained for 56 patients, which constitute the subgroup with extended echocardiographic follow‐up (Figure 1). Compared to the overall population, patients with an extended follow‐up were significantly younger, had a significantly lower STS risk score and had a significantly lower incidence of New York Heart Association (NYHA) class III/IV before TAVI (Supporting Information S3: Table 1).

### 3.2 Long‐Term Clinical Outcomes

**Figure 2:**
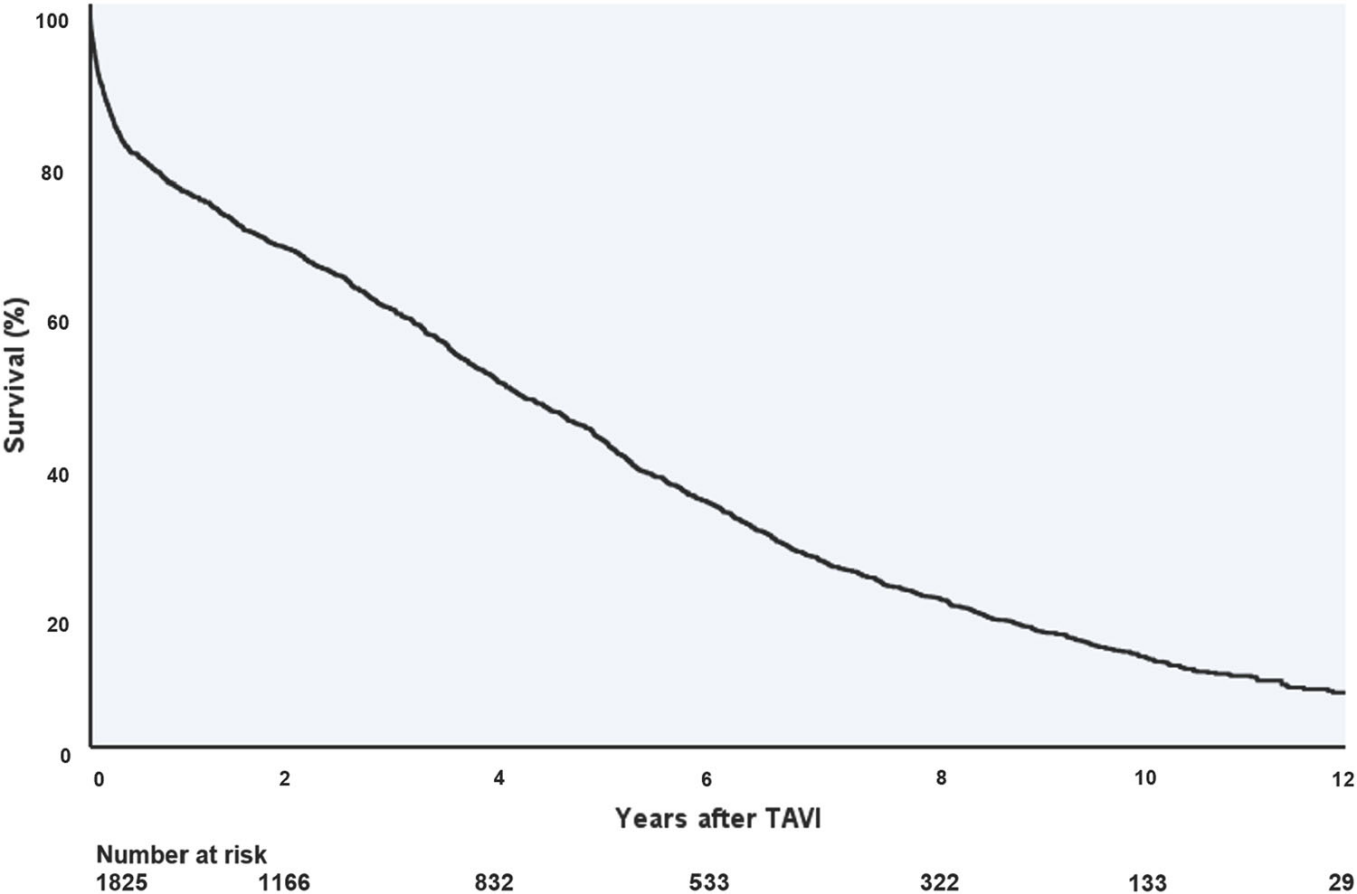
Kaplan‐Meier curve of the overall survival of the total study population. Survival of the overall population, calculated by Kaplan–Meier estimates. TAVI = transcatheter aortic valve implantation. [Color figure can be viewed at wileyonlinelibrary.com]

The median survival of the overall population was 1399 days. After 1‐, 3‐, 5‐, 7‐, 9‐, 10‐, 11‐, and 12 years, all‐cause mortality rates were 26.0%, 42.2%, 59.9%, 75.1%, 84.0%, 87.6%, 90.6%, and 92.8%, respectively (Figure 2).

**Figure 3:**
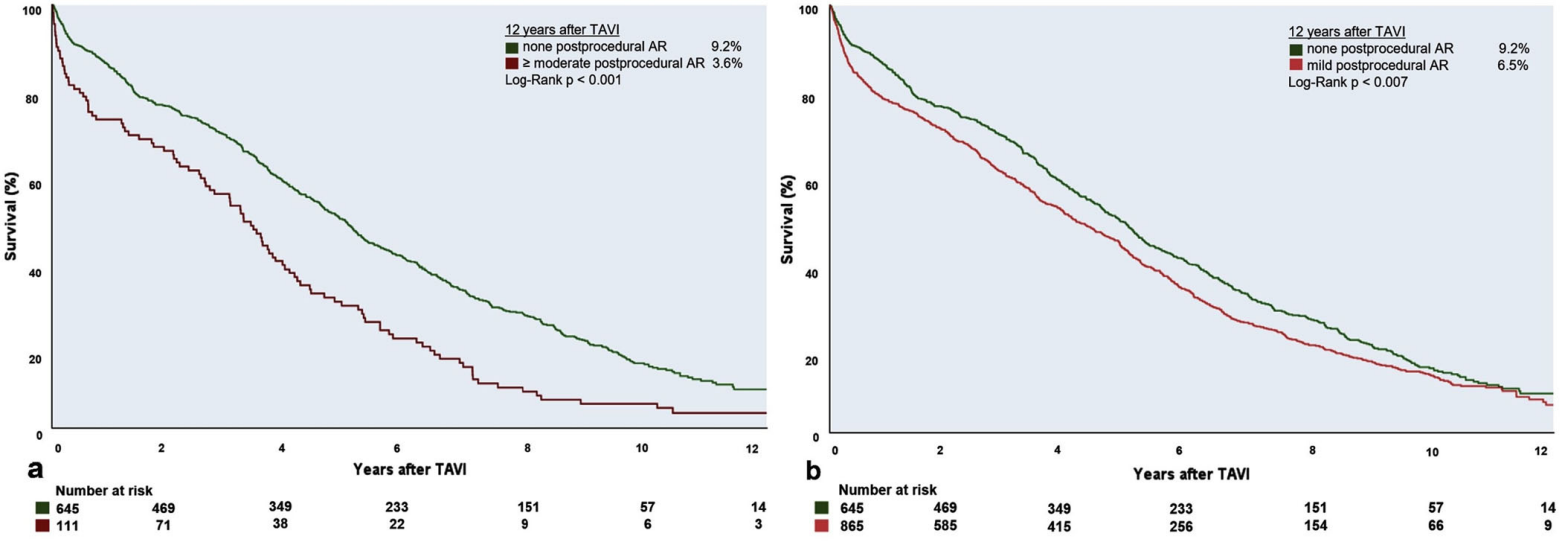
Survival stratified by the presence or absence of post‐procedural AR. Kaplan–Meier curves comparing mortality rates of patients with no versus more than mild postprocedural aortic regurgitation (a) and no versus mild postprocedural aortic regurgitation (b). AR = aortic regurgitation. [Color figure can be viewed at wileyonlinelibrary.com]

In the Kaplan–Meier‐analysis, patients with postprocedural AR ≥ 2 showed a significantly higher mortality than patients with no postprocedural AR (median survival of 3.2 years vs. 4.8 years, *p* < 0.001, Figure 3a). The impact on mortality persisted after 1‐year in a landmark analysis (Supporting Information S1: Figure 1a). Patients with mild post‐procedural AR also showed a significantly higher mortality compared to patients with no post‐procedural AR (median survival of 4.1 vs. 4.8 years, *p* = 0.007, Figure 3b), but this association was only observed during the first postprocedural year in a landmark analysis (Supporting Information S1: Figure 1b). Survival was also higher in patients receiving SE THVs than in patients treated with BE THVs (4.1 years vs. 3.5 years, *p* = 0.028, Supporting Information S2: Figure 2), but this effect diminished when incorporating valve type in a multivariable Cox‐regression model.

Overall, independent predictors of mortality included age (hazard ratio [HR] 1.02, 95% confidence interval [CI]: 1.01–1.03, *p* < 0.001), STS score (HR 1.02, 95% CI: 1.01–1.03, *p* < 0.001), baseline NYHA Class III or IV (HR 1.41, 95% CI: 1.22–1.63, *p* < 0.001), diabetes mellitus (HR 1.28, 95% CI: 1.14–1.44, *p* < 0.001), peripheral arterial disease (HR 1.57, 95% CI: 1.34–1.84, *p* < 0.001), chronic renal dialysis (HR 2.06, 95% CI: 1.52–2.79, *p* < 0.001), moderate postprocedural AR (HR 1.74 CI: 1.40–2.17, *p* < 0.001) and mild postprocedural AR (HR 1.20 CI: 1.06–1.35), as listed in Table 3. The majority of patients (54.3%) presented in NYHA class I/II at long‐term follow‐up.

**Table 3:** Multivariate cox regression analysis for predictors of mortality for the total population.

	Univariate	Multivariate	
	*p* value	HR (95% CI)	*p* value
Age, years	< 0.001	1.02 (1.008–1.029)	< 0.001
Weight, kg	0.028	1.00 (0.995–1.003)	0.684
STS score, %	< 0.001	1.02 (1.014–1.032)	< 0.001
NYHA class III/IV	< 0.001	1.41 (1.218–1.627)	< 0.001
Diabetes mellitus	< 0.001	1.28 (1.137–1.437)	< 0.001
Peripheral arterial disease	< 0.001	1.57 (1.340–1.843)	< 0.001
Pulmonary hypertension	0.028	1.04 (0.919–1.171)	0.555
Chronic renal dialysis	< 0.001	2.06 (1.520–2.789)	< 0.001
Type of THV	0.028	1.05 (0.929–1.175)	0.467
Mild postprocedural AR	< 0.001	1.20 (1.059–1.348)	0.004
Moderate postprocedural AR	< 0.001	1.74 (1.398–2.169)	< 0.001

Abbreviations: AR, aortic regurgitation; NYHA, New York Heart Association; STS score, Society of Thoracic Surgeons Score; THV, transcatheter heart valve.

### 3.3 Long‐Term Echocardiographic Outcomes

Echocardiographic data after 1‐, 3‐, 5‐, 7‐, 9‐, 10‐, 11‐, and 12 years were available for 922, 351, 180, 90, 37, 16, 4, and 5 patients, respectively. There was a full long‐term echocardiographic follow‐up at ≥ 9 years for 56 patients. Median echocardiographic follow‐up of this subgroup was 3589 days, with the longest echocardiographic follow‐up reaching 4661 days. Annual echocardiographic data of the total and the extended follow‐up population are presented in Supporting Information S3: Tables 2 and 3.

In the extended follow‐up cohort, there were numerical but statistically nonsignificant changes between the time of discharge and follow‐up in Pmax, Pmean and in the incidence of ≥ moderate AR for both THVs, as illustrated in Supporting Information S3: Table 4.

At long‐term follow‐up, Pmax, and Pmean in BE valves were significantly higher than in SE valves, and the EOA was significantly smaller, as presented in Table 4.

**Table 4:** Comparison of echocardiographic parameters between SE THVs and BE THVs within the extended follow‐up cohort at the timepoint of discharge and at the time of follow‐up.

	Timepoint of TAVI	Self‐expanding THV (*n* = 36)	Balloon‐expandable THV (*n* = 20)	*p* value
LVEF, %	At discharge	61.0 [54.3; 66.0]	59.6 [51.0; 68.7]	0.864
	At long‐term follow‐up	54.0 [4.3; 60.8]	54.0 [48.5; 58.0]	0.777
EOA, cm^2^	At discharge	2.0 [1.7; 2.6]	1.7 [1.2; 2.1]	0.055
	At long‐term follow‐up	1.7 [1.4; 2.1]	1.4 [1.2; 1.5]	0.008
Pmean, mmHg	At discharge	7.4 [5.2; 10.9]	8.7 [6.1; 11.4]	0.446
	At long‐term follow‐up	6.0 [5.0; 10.0]	10.0 [6.0; 17.0]	0.017
Pmax, mmHg	At discharge	15.5 [10.0; 20.4]	15.9 [12.1; 21.7]	0.478
	At long‐term follow‐up	12.0 [9.0; 18.8]	21.0 [14.5; 31.0]	0.004
≥ moderate AR	At discharge	1 (2.8%)	1 (5.0%)	0.668
	At long‐term follow‐up	3 (8.3%)	3 (15.0%)	0.440
	‐transvalvular	0	2	
	‐paravalvular	3	1	
any SVD	At long‐term follow‐up	1 (2.8%)	4 (20%)	0.030

*Note:* Values are *n* (%), mean ± standard deviation or median [interquartile range].

Abbreviations: AR, aortic regurgitation; EOA, effective orifice area; LVEF, left ventricular ejection fraction; Pmax, peak transvalvular pressure gradient; Pmean, mean transvalvular pressure gradient; SVD, structural valve deterioration; TAVI, transcatheter aortic valve implantation; THV, Transcatheter heart valve.

### 3.4 THV Durability

**Figure 4:**
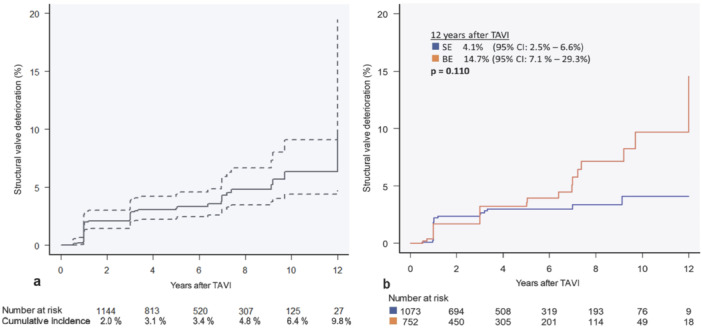
Cumulative incidence of SVD. Graphical illustration of the cumulative incidence of SVD of the total population (a) and as a comparison for SE and BE THV (b), adjusted for mortality. BE = balloon‐expandable, CI = confidence interval, SE = self‐expanding, SVD = structural valve deterioration, THV = transcatheter heart valve. [Color figure can be viewed at wileyonlinelibrary.com]

In the overall population, any SVD was observed in 51 patients. The cumulative incidence of SVD adjusted to all‐cause mortality of the total population was 1.8% (95% CI: 1.20%–2.65%) after 1 year, 2.7% (95% CI: 1.92%–3.74%) at 3 years, 3.2% (95% CI: 2.36%–4.42%) at 5 years, 4.3% (95% CI: 3.12%–5.94%) at 7 years, 4.8% (95% CI: 3.50%–6.68%) at 9 years, 6.4% (95% CI: 4.41%–9.12%) at 10 years, 6.4% (95% CI: 4.41%–9.12%) at 11 years, and 9.8% (95% CI: 4.75%–19.48%) at 12 years (Figure 4a).

There was a trend toward a lower incidence of SVD at 12 years in patients treated with SE valves compared to BE valves (4.1%, 95% CI: 2.52%–6.69% vs. 14.7%, 95% CI: 7.07%–29.32%, *p* = 0.110), although the difference was not statistically significant (Figure 4b).

Within the subpopulation with the extended follow‐up, any SVD occurred significantly less frequently with SE valves compared to BE valves (2.8% vs. 20.0%, *p* = 0.030). Severe SVD occurred in two patients. One patient had a 26 mm BE valve implanted in 2009 through the transapical approach. Pmean increased from 9.2 mmHg at discharge to 50.0 mmHg at 12.5 years after TAVI. The second patient was treated with a 31 mm SE prothesis through a transfemoral access in 2012. In this patient, there was an increase in Pmean from 12.0 to 37.0 mmHg after 9.1 years together with an additional decrease in EOA from 2.1 to 0.7 cm^2^. Moderate SVD was observed in three patients. One patient, treated with a 26 mm BE valve in 2009, had an increase in Pmean from 14.0 to 26.0 mmHg after 12 years. The other two patients, also initially treated with BE valves, suffered from new‐onset moderate transprosthetic AR after 10.2 and 9.7 years.

## 4 Discussion

The aim of this study was to evaluate long‐term mortality and durability of THVs implanted during the early TAVI experience at a single high‐volume center in Germany. The main findings can be summarized as follows: (1) the overall study population showed a very high 12‐year mortality rate of 92.8%, which is nevertheless in line with the average life expectancy of octogenarians; (2) moderate postprocedural AR was associated with a significantly higher mortality at long‐term follow‐up; (3) the rate of any SVD was low, with better long‐term hemodynamic function of SE valves compared with BE valves; (4) patients who survived up to 12 years presented in a favorable functional status and had largely preserved valve function.

Data evaluating the long‐term durability of TAVI remain scarce, mainly due to the low survival rates over an extended follow‐up period within the predominantly elderly population treated with TAVI in the past two decades. Within the current study, the patient population presented with numerous comorbidities at baseline, which largely contributed to the high mortality rates observed during the follow‐up period. These outcomes align with previous studies reporting a 5‐year mortality rate of 59.0% [19] and an 8‐ and 10‐year‐mortality rate of 73.0% and 91.6%, respectively [12], which emphasizes the challenge of having only a small fraction of the initial population available for long‐term analysis of THV function. Nevertheless, it is noteworthy that the mortality rates observed in this study do not markedly differ from the general life expectancy of octogenarian [20].

One important consideration following TAVI is the presence of paravalvular AR. Numerous studies have highlighted the negative impact of residual AR, particularly when it is considered moderate or severe, on both short‐ [21, 22] and long‐term survival [18]. However, the effect of mild AR on mortality remains controversial. While some studies did not detect an association between mild AR and mortality [23, 24], more recent ones with more granular assessment of AR have demonstrated a significant association between mild AR and all‐cause and cardiovascular mortality [21, 22, 25, 26, 27], which is consistent with the findings of the current study. Possible mechanisms contributing to the negative impact of residual AR may include persistently increased enddiastolic volumes, unfavorable left ventricular remodeling [28], and a potentially higher incidence of bleeding [27], which may all be possible consequences even in mild AR. However, the reproducibility of this association remains questionable due the largely semiquantitative approach for AR assessment applied in our study and the utilization of different grading systems for AR quantification that may impact comparability [29].

In this registry, survival was affected by the type of THV in the unadjusted analysis, as patients treated with SE THV had a significantly longer survival compared with patients treated with BE THV. However, this effect disappeared in the adjusted analysis. Whether THV type may influence long‐term survival remains a matter of debate, which is reflected by varying results in previous studies ranging from better survival with SE THVs [30] to longer survival with BE THVs [31] or no association between THV type and mortality [19, 32]. Two recently published studies involving a low‐risk patient population should be highlighted in this context: the Evolut Low‐Risk trial, which compared 4‐year outcomes between SAVR and SE THV [33], and the PARTNER‐3 low risk trial that analyzed 5‐year outcomes in both BE‐TAVI and SAVR [34]. It is important to note that a direct comparison between these two randomized trials is not feasible. A more comprehensive evaluation of the impact of THV type on mortality necessitates a longer follow‐up period and randomized studies comparing both types of THV, particularly in low‐risk patients. Nonetheless, the results of both trials are noteworthy. In the PARTNER‐3, no significant difference was observed in the primary endpoint including all‐cause mortality between BE‐TAVI and SAVR. In contrast, the Evolut Low‐Risk trial reported lower mortality and superior hemodynamic function in patients treated with SE‐TAVI compared to SAVR. In the current study, additional predictors of mortality were identified, but these were generally known risk factors for mortality, such as cardiovascular comorbidities or increasing age.

The current study represents the largest cohort of patients with an echocardiographic follow‐up and durability analysis beyond 10 years in Europe, thus contributing valuable insights within a field of very limited knowledge but a potentially important impact on lifetime management in patients with aortic stenosis. Echocardiographic evaluation of patients who survived more than 9 years revealed favorable valve function in the majority of cases, which is consistent with previous findings [12, 35]. The overall population had a cumulative incidence for any SVD of 6.4% at 10 years and 9.8% at 12 years after TAVI. These results are comparable with previously published data for long‐term results after SAVR [5] and are well aligned with 10‐year post TAVI findings of previous small studies that reported a cumulative incidence for any SVD of 4.7% in 6 long‐term survivors [13], 6.5% in a small cohort of 19 high‐risk survivors [12] and 15.4% in the recently published NOTION trial, that provided 10‐year echocardiographic follow‐up for 36 low‐risk patients that were compared with SAVR [11].

Despite the favorable hemodynamic function of both SE and BE THVs, it is noteworthy to emphasize the trend toward superior long‐term durability for SE THVs compared with BE valves in the overall population (SVD 4.1% vs. 14.7%, *p* = 0.110), and the significant difference favoring SE valves in the extended follow‐up cohort. BE valves showed a significantly lower EOA and higher transvalvular gradients at long‐term follow‐up, which are pivotal components in defining SVD. These findings support previous results from a randomized trial, which demonstrated a significantly lower mean gradient and a significantly higher EOA in SE valves compared to BE valves after a period of 5 years [36]. Differences between both platforms can be further explained by procedural differences such as the mechanism of deployment, the tendency to oversizing and the supraannular design of SE valves, but this still needs to be confirmed with newer generation valves in a larger series of patients. Overall, the influence of THV type on durability, as well as its association with mortality, requires more comprehensive assessment and further evaluation.

### 4.1 Study Limitations

Although our study provides the currently largest population with very long‐term follow‐up after TAVI, it has several important limitations. First, the present publication is a single‐center retrospective study, with its obvious limitations. Second, we included patients who underwent TAVI from 2006 to 2012. Over the past decade, continuous advancements have been made in valve technology, diagnostic work‐up and the TAVI procedure itself. This may prevent the generalization of the results to current generation THVs. Third, despite adhering to the latest VARC‐3 recommendations, SVD over‐ and underestimation is conceivable due to a substantial number of patients lost to follow‐up. This could have been exacerbated by the high mortality rate observed in an elderly high‐risk population, though a competing risk analysis was performed to account for this limitation. Fourth, the COVID19 pandemic markedly impacted our on‐site clinical and echocardiographic follow‐up. Government‐imposed restrictions and safety regulations limited visits to public institutions to urgent purposes (hospitals, outpatient clinics and others). Consequently, many patients declined attending follow‐up examinations at our center out of concern for their own health. Factors such as multimorbidity and lack of mobility further hindered elderly patients from attending the offered follow‐up visits. In the absence of regularly scheduled long‐term follow‐up visits after the procedure, only limited echocardiographic follow‐up data was available. Fifth, the study does not provide data on reintervention and valve‐related mortality, which can be attributed to the low number of post‐mortem analyses conducted in Germany and the high rate of patients lost to follow‐up. Finally, the lack of a standardized protocol for echocardiographic assessment, coupled with inclusion of results from examinations of referring cardiologists, may have caused variability in measured echocardiographic parameters. Nevertheless, this study includes a large patient cohort and one of the largest post‐TAVI datasets in Europe beyond 8 years with echocardiographic data of up to 12 years.

## 5 Conclusions

In a population of TAVI patients treated at the Heart Center Leipzig at Leipzig University between 2006 and 2012, the 12‐year mortality was 92.8%. The cumulative incidence of SVD was 9.8% at 12 years, and SE valves appear to have better durability compared with BE valves. Patients surviving at long‐term presented in a favorable functional status with good valve function.

## Conflicts of Interest

N. Magner received a research and an educational grant from Abiomed to his institution, outside the submitted work; an educational grant from Boston Scientific to his institution, outside the submitted work; received personal fees from Edwards Lifesciences, Medtronic, Biotronik, Novartis, Sanofi Genzyme, AstraZeneca, Pfizer, Daiichi Sankyo, Abbott, Abiomed, B. Braun, and Boston Scientific, outside the submitted work. M. Abdel‐Wahab declares that his hospital receives speaker's honoraria and/or consultancy fees on his behalf from Boston Scientific and Medtronic. The other authors declare no conflicts of interest.

## Supporting information

The Supplemtary.

The Supplemtary.

The Supplemtary.

## References

[1] J. Joseph , S. Y. Naqvi , J. Giri , and S. Goldberg , “Aortic Stenosis: Pathophysiology, Diagnosis, and Therapy,” American Journal of Medicine 130 (2017): 253–263, 10.1016/j.amjmed.2016.10.005. 27810479

[2] B. H. Grimard , R. E. Safford , and E. L. Burns., “Aortic Stenosis: Diagnosis and Treatment,” American Family Physician 93 (2016): 371–378. 26926974

[3] F. Beyersdorf , A. Vahanian , M. Milojevic , et al., “2021 ESC/EACTS Guidelines for the Management of Valvular Heart Disease,” European Journal of Cardio‐Thoracic Surgery 60 (2021): 727–800, 10.1093/ejcts/ezab389. 34453161

[4] T. J. Cahill , M. Chen , K. Hayashida , et al., “Transcatheter Aortic Valve Implantation: Current Status and Future Perspectives,” European Heart Journal 39 (2018): 2625–2634, 10.1093/eurheartj/ehy244. 29718148

[5] T. Rodriguez‐Gabella , P. Voisine , F. Dagenais , et al., “Long‐Term Outcomes Following Surgical Aortic Bioprosthesis Implantation,” Journal of the American College of Cardiology 71 (2018): 1401–1412, 10.1016/j.jacc.2018.01.059. 29598859

[6] P. Pibarot , J. Ternacle , W. A. Jaber , et al., “Structural Deterioration of Transcatheter Versus Surgical Aortic Valve Bioprostheses in the PARTNER‐2 Trial,” Journal of the American College of Cardiology 76 (2020): 1830–1843, 10.1016/j.jacc.2020.08.049. 33059828

[7] R. R. Makkar , V. H. Thourani , M. J. Mack , et al., “Five‐Year Outcomes of Transcatheter or Surgical Aortic‐Valve Replacement,” New England Journal of Medicine 382 (2020): 799–809, 10.1056/NEJMoa1910555. 31995682

[8] S. L. Swift , T. Puehler , K. Misso , et al., “Transcatheter Aortic Valve Implantation Versus Surgical Aortic Valve Replacement in Patients With Severe Aortic Stenosis: A Systematic Review and Meta‐Analysis,” BMJ Open 11 (2021): e054222, 10.1136/bmjopen-2021-054222. PMC8650468 34873012

[9] T. H. Jørgensen , H. G. H. Thyregod , N. Ihlemann , et al., “Eight‐Year Outcomes for Patients With Aortic Valve Stenosis at Low Surgical Risk Randomized to Transcatheter vs. Surgical Aortic Valve Replacement,” European Heart Journal 42 (2021): 2912–2919, 10.1093/eurheartj/ehab375. 34179981 PMC8347457

[10] L. Søndergaard , N. Ihlemann , D. Capodanno , et al., “Durability of Transcatheter and Surgical Bioprosthetic Aortic Valves in Patients at Lower Surgical Risk,” Journal of the American College of Cardiology 73 (2019): 546–553, 10.1016/j.jacc.2018.10.083. 30732707

[11] H. G. H. Thyregod , T. H. Jørgensen , N. Ihlemann , et al., “Transcatheter or Surgical Aortic Valve Implantation: 10‐Year Outcomes of the Notion Trial,” European Heart Journal 45 (2024): 1116–1124, 10.1093/eurheartj/ehae043. 38321820 PMC10984572

[12] J. Sathananthan , S. Lauck , J. Polderman , et al., “Ten Year Follow‐Up of High‐Risk Patients Treated During the Early Experience With Transcatheter Aortic Valve Replacement,” Catheterization and Cardiovascular Interventions: Official Journal of the Society for Cardiac Angiography & Interventions 97 (2021): 431, 10.1002/ccd.29124. 32940418

[13] J. Stehli , M. Dagan , S. J. Duffy , et al., “Long‐Term Valve Durability in Patients Undergoing Transcatheter Aortic Valve Implantation,” Heart, Lung & Circulation 32 (2023): 240–246, 10.1016/j.hlc.2022.10.006. 36376193

[14] C. R. Smith , M. B. Leon , M. J. Mack , et al., “Transcatheter Versus Surgical Aortic‐Valve Replacement in High‐Risk Patients,” New England Journal of Medicine 364 (2011): 2187–2198, 10.1056/NEJMoa1103510. 21639811

[15] D. H. Adams , J. J. Popma , M. J. Reardon , et al., “Transcatheter Aortic‐Valve Replacement With a Self‐Expanding Prosthesis,” New England Journal of Medicine 370 (2014): 1790–1798, 10.1056/NEJMoa1400590. 24678937

[16] M. Abdel‐Wahab , F. J. Neumann , J. Mehilli , et al., “1‐Year Outcomes After Transcatheter Aortic Valve Replacement With Balloon‐Expandable Versus Self‐Expandable Valves,” Journal of the American College of Cardiology 66 (2015): 791–800, 10.1016/j.jacc.2015.06.026. 26271061

[17] M. Abdel‐Wahab , J. Mehilli , C. Frerker , et al., “Comparison of Balloon‐Expandable vs Self‐Expandable Valves in Patients Undergoing Transcatheter Aortic Valve Replacement: The Choice Randomized Clinical Trial,” Journal of the American Medical Association 311 (2014): 1503–1514, 10.1001/jama.2014.3316. 24682026

[18] P. Généreux , N. Piazza , M. C. Alu , et al., “Valve Academic Research Consortium 3: Updated Endpoint Definitions for Aortic Valve Clinical Research,” European Heart Journal 42 (2021): 1825–1857, 10.1093/eurheartj/ehaa799. 33871579

[19] S. Haussig , C. Pleissner , N. Mangner , et al., “Long‐Term Follow‐Up After Transcatheter Aortic Valve Replacement,” CJC Open 3 (2021): 845–853, 10.1016/j.cjco.2021.01.012. 34401691 PMC8347830

[20] Statistisches Bundesamt (Destatis) . “Durchschnittliche Lebenserwartung (Periodensterbetafel): Deutschland, Jahre, Geschlecht, Vollendetes Alter [Average Life Expectancy (Period Life Table): Germany, Years, Sex, Completed Age]” (2024), https://www-genesis.destatis.de/datenbank/online/url/5acc3d64.

[21] S. Kodali , P. Pibarot , P. S. Douglas , et al., “Paravalvular Regurgitation After Transcatheter Aortic Valve Replacement With the Edwards Sapien Valve in the Partner Trial: Characterizing Patients and Impact on Outcomes,” European Heart Journal 36 (2015): 449–456, 10.1093/eurheartj/ehu384. 25273886

[22] S. H. Little , J. K. Oh , L. Gillam , et al., “Self‐Expanding Transcatheter Aortic Valve Replacement Versus Surgical Valve Replacement in Patients at High Risk for Surgery: A Study of Echocardiographic Change and Risk Prediction,” Circulation: Cardiovascular Interventions 9 (2016): e003426, 10.1161/CIRCINTERVENTIONS.115.003426. 27313280

[23] E. van Belle , F. Juthier , S. Susen , et al., “Postprocedural Aortic Regurgitation in Balloon‐Expandable and Self‐Expandable Transcatheter Aortic Valve Replacement Procedures: Analysis of Predictors and Impact on Long‐Term Mortality: Insights From the FRANCE2 Registry,” Circulation 129 (2014): 1415–1427, 10.1161/CIRCULATIONAHA.113.002677. 24566199

[24] N. Yoshijima , R. Yanagisawa , H. Hase , et al., “Update on the Clinical Impact of Mild Aortic Regurgitation After Transcatheter Aortic Valve Implantation: Insights From the Japanese Multicenter OCEAN‐TAVI Registry,” Catheterization and Cardiovascular Interventions 95 (2020): 35–44, 10.1002/ccd.28279. 30977256

[25] T. Ando , A. Briasoulis , T. Telila , L. Afonso , C. L. Grines , and H. Takagi , “Does Mild Paravalvular Regurgitation Post Transcatheter Aortic Valve Implantation Affect Survival? A Meta‐Analysis,” Catheterization and Cardiovascular Interventions 91 (2018): 135–147, 10.1002/ccd.27336. 28963761

[26] B. M. Jones , E. M. Tuzcu , A. Krishnaswamy , et al., “Prognostic Significance of Mild Aortic Regurgitation in Predicting Mortality After Transcatheter Aortic Valve Replacement,” Journal of Thoracic and Cardiovascular Surgery 152 (2016): 783–790, 10.1016/j.jtcvs.2016.05.023. 27321435

[27] T. Okuno , D. Tomii , D. Heg , et al., “Five‐Year Outcomes of Mild Paravalvular Regurgitation After Transcatheter Aortic Valve Implantation,” EuroIntervention 18 (2022): 33–42, 10.4244/EIJ-D-21-00784. 34930717 PMC9904370

[28] K. C. Chau , S. Chen , A. Crowley , et al., “Paravalvular Regurgitation After Transcatheter Aortic Valve Replacement in Intermediate‐Risk Patients: A Pooled Partner 2 Study,” EuroIntervention 17 (2022): 1053–1060, 10.4244/EIJ-D-20-01293. 34483095 PMC9724907

[29] P. Lancellotti , N. Piazza , and T. Modine , “Quantification of Paravalvular Regurgitation After Transcatheter Aortic Valve Implantation: Improved Accuracy Means Better Standardization,” European Heart Journal – Cardiovascular Imaging 17 (2016): 861–862, 10.1093/ehjci/jew110. 27252487

[30] V. M. Collas , C. Dubois , V. Legrand , et al., “Midterm Clinical Outcome Following Edwards Sapien or Medtronic Corevalve Transcatheter Aortic Valve Implantation (TAVI): Results of the Belgian TAVI Registry,” Catheterization and Cardiovascular Interventions 86 (2015): 528–535, 10.1002/ccd.25999. 25963917

[31] G. Costa , P. D'Errigo , S. Rosato , et al., “Long‐Term Outcomes of Self‐Expanding Versus Balloon‐Expandable Transcatheter Aortic Valves: Insights From the OBSERVANT Study,” Catheterization and Cardiovascular Interventions 98 (2021): 1167–1176, 10.1002/ccd.29701. 33847447

[32] A. Gasecka , M. Walczewski , A. Witkowski , et al., “Long‐Term Mortality After TAVI for Bicuspid vs. Tricuspid Aortic Stenosis: A Propensity‐Matched Multicentre Cohort Study,” Frontiers in Cardiovascular Medicine 9 (2022): 894497, 10.3389/fcvm.2022.894497. 35800165 PMC9253589

[33] J. K. Forrest , G. M. Deeb , S. J. Yakubov , et al., “4‐Year Outcomes of Patients With Aortic Stenosis in the Evolut Low Risk Trial,” Journal of the American College of Cardiology 82 (2023): 2163–2165, 10.1016/j.jacc.2023.09.813. 37877907

[34] M. J. Mack , M. B. Leon , V. H. Thourani , et al., “Transcatheter Aortic‐Valve Replacement in Low‐Risk Patients at Five Years,” New England Journal of Medicine 389 (2023): 1949–1960, 10.1056/NEJMoa2307447. 37874020

[35] M. Barbanti , G. Costa , P. Zappulla , et al., “Incidence of Long‐Term Structural Valve Dysfunction and Bioprosthetic Valve Failure After Transcatheter Aortic Valve Replacement,” Journal of the American Heart Association 7 (2018): e008440, 10.1161/JAHA.117.008440. 30371244 PMC6201462

[36] M. Abdel‐Wahab , M. Landt , F. J. Neumann , et al., “5‐Year Outcomes After TAVR With Balloon‐Expandable Versus Self‐Expanding Valves,” JACC: Cardiovascular Interventions 13 (2020): 1071–1082, 10.1016/j.jcin.2019.12.026. 32305398

