# Supplementary figures and images for: Outcomes Beyond 10 Years After Transcatheter Aortic Valve Implantation in High‐Risk Patients With Severe Aortic Valve Stenosis
Source: Catheter Cardiovasc Interv. 2025 Jun 12;106(2):1301–9. doi: 10.1002/ccd.31677 (PMC12336767; doi:10.1002/ccd.31677)

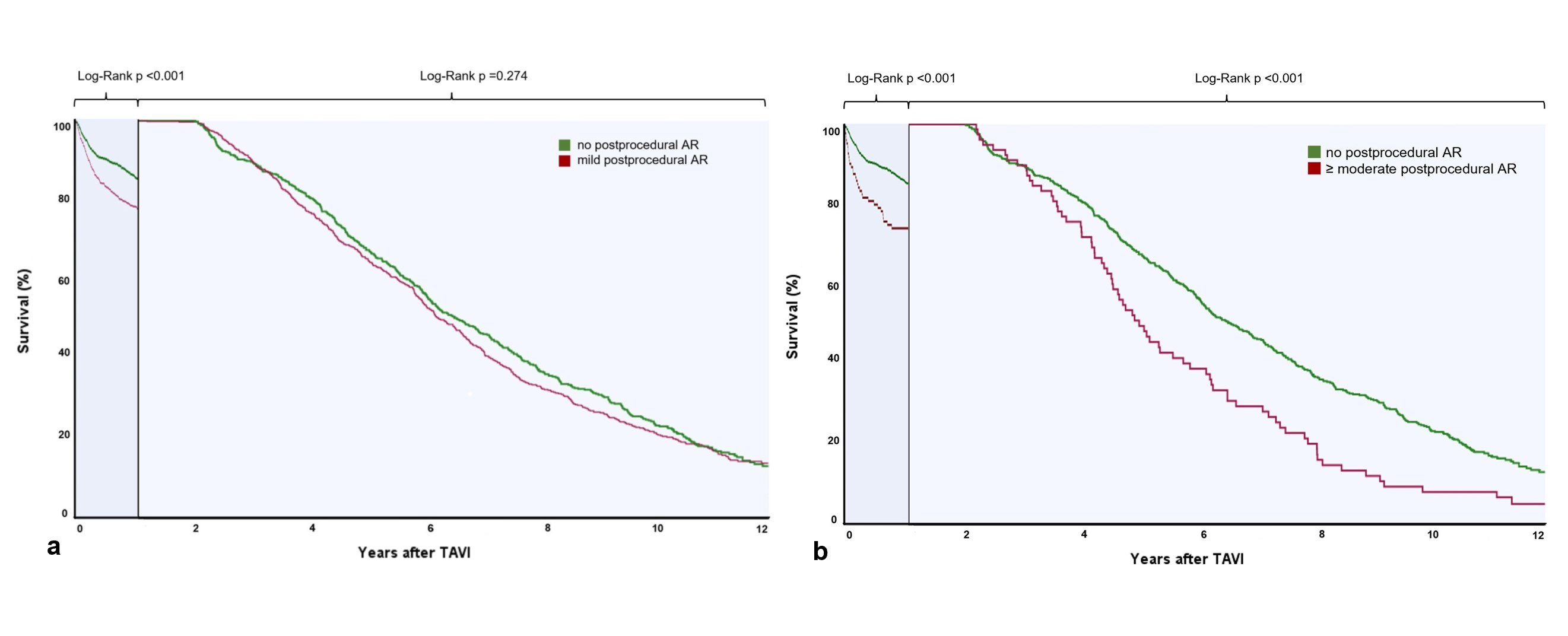

Supplement: Supplementary file 1 — The Supplemtary. [file CCD-106-1301-s001.tif]

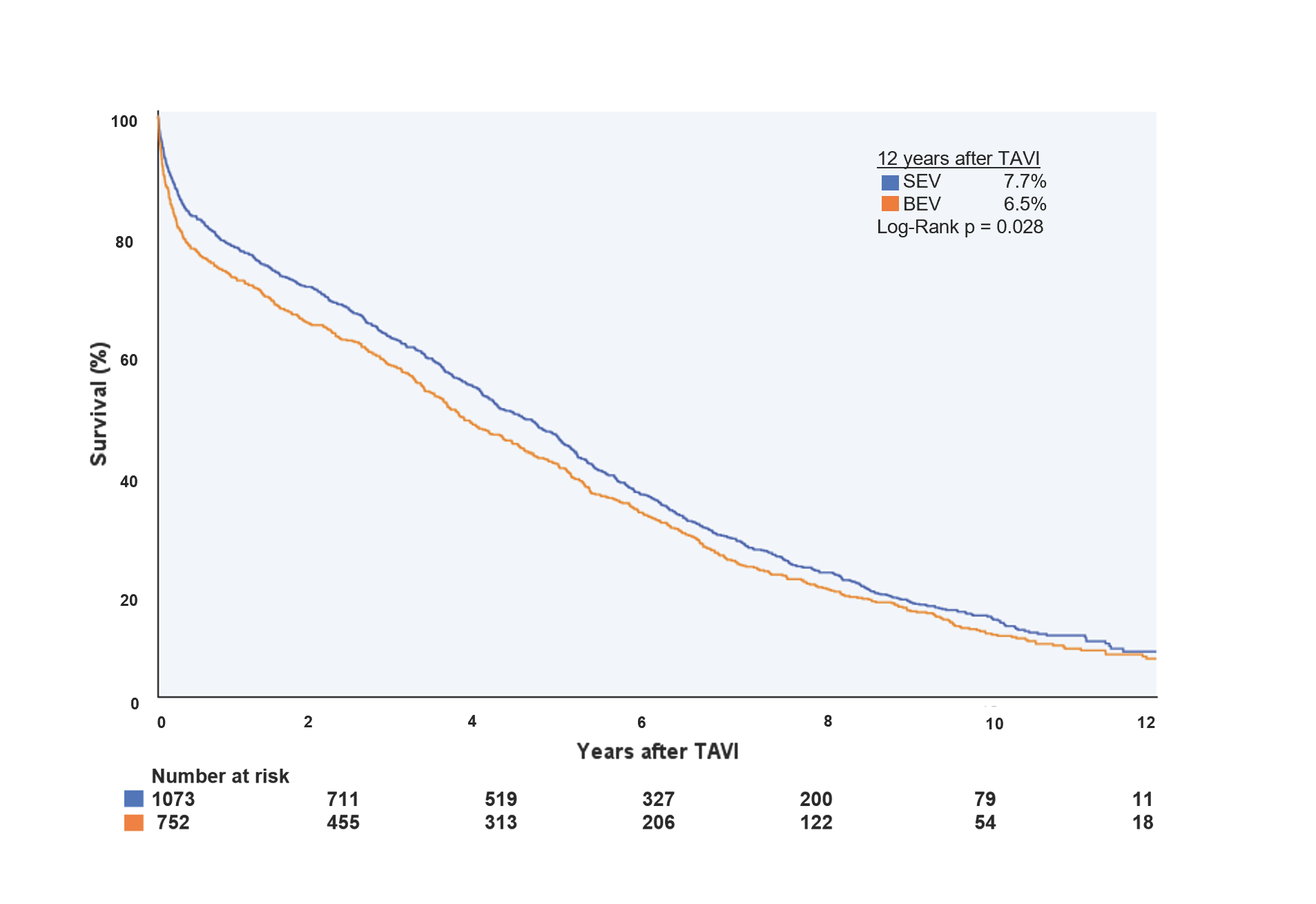

Supplement: Supplementary file 2 — The Supplemtary. [file CCD-106-1301-s002.tif]
