# Supplementary material for: Outcomes Beyond 10 Years After Transcatheter Aortic Valve Implantation in High‐Risk Patients With Severe Aortic Valve Stenosis
Source: Catheter Cardiovasc Interv. 2025 Jun 12;106(2):1301–9. doi: 10.1002/ccd.31677 (PMC12336767; doi:10.1002/ccd.31677)
Supplement: Supplementary file 3 — The Supplemtary. [file CCD-106-1301-s003.docx]

**Supplement**

**Supplementary tables**

**Supplentary table 1 –** Comparison of baseline characteristics and echocardiographic parameters at baseline and at discharge between the extended follow-up cohort and the remaining study population

|  | Follow-up cohort  (n=56) | Remaining study population  (n=1769) | p-value |
| --- | --- | --- | --- |
| Baseline characteristics |  |  |  |
| Age, years | 79.0 [73.3; 82.0] | 81.0 [77.0;85.0] | <0.001 |
| Female | 27 (48.2%) | 1053 (59.5%) | 0.090 |
| STS Score | 3.9 [3.1;6.2] | 7.6 [4.9; 12.0] | <0.001 |
| Hypertension | 52 (92.9%) | 1675 (94.7%) | 0.550 |
| Diabetes mellitus | 24 (42.9%) | 812 (45.9%) | 0.653 |
| Coronary artery disease | 25 (44.6%) | 922 (52.1%) | 0.270 |
| Carotid stenosis | 14 (25.0%) | 312 (17.6%) | 0.157 |
| Peripheral arterial disease | 5 (8.9%) | 240 (13.6%) | 0.316 |
| Previous stroke | 9 (16.1%) | 175 (9.9%) | 0.131 |
| Previous myocardial infarction | 10 (17.9%) | 237 (13.7%) | 0.374 |
| Pulmonary hypertension | 19 (33.9%) | 595 (33.6%) | 0.963 |
| Chronic renal dialysis | 0 (0.0%) | 64 (3.6%) | 0.147 |
| NYHA class III/IV | 34 (60.7%) | 1434 (81.1%) | <0.001 |
| Echocardiographic parameters at baseline | | |  |
| LVEF, % | 63.0  [52.7;68.5] | 58.0  [46.0; 65.0] | 0.080 |
| EOA, cm² | 0.7  [0.5; 0.8] | 0.6  [0.5;0.8] | 0.181 |
| Pmean, mmHg | 43.0  [35.8; 53.0] | 44.0  [34.0; 55.7] | 0.669 |
| Pmax, mmHg | 70.0  [56.9; 85.0] | 70.7  [55.8;87.0] | 0.837 |
| ≥ moderate AR | 10 (17.9%) | 309 (17.5%) | 0.917 |
| Echocardiographic parameters at discharge | | |  |
| LVEF, % | 60.0  [54.0; 67.1] | 60.0  [50.0; 65.0] | 0.190 |
| EOA, cm² | 1.9  [1.5; 2.2] | 1.7  [1.4; 2.1] | 0.018 |
| Pmean, mmHg | 7.9  [5.9; 10.9] | 9.0  [6.2; 12.0] | 0.114 |
| Pmax, mmHg | 15.90  [11.0; 20.5] | 16.1  [12.0; 22.0] | 0.290 |
| ≥ moderate AR | 2 (3.6%) | 109 (5.6%) | 0.324 |
| Values are n (%), mean ± standard deviation or median [interquartile range]  AR, aortic regurgitation; EOA, effective orifice area; LVEF, left ventricular ejection fraction; NYHA New York Heart Association; Pmax, Peak transvalvular pressure gradient; Pmean, mean transvalvular pressure gradient; STS Score, Society of Thoracic Surgeons Score | | | |

**Supplementary table 2** – Echocardiographic parameters over time of the total study population

|  | 1 year | 3 years | 5 years | 7 years | 9 years | 10 years | 11 years | 12 years |
| --- | --- | --- | --- | --- | --- | --- | --- | --- |
| Total population (n=1825) | n=922 | n=351 | n=180 | n=90 | n =37 | n = 16 | n = 4 | n=5 |
| LVEF, % | 59.0  [51.0;  65.0] | 58.0  [50.0;  64.3] | 56.0  [49.0; 61.3] | 55.0  [46.0; 60.0] | 52.0  [49.0; 57.0] | 54.5  [48.3; 63.8] | 55.5  [27.8; 63.0] | 61.0  [49.0; 65.5] |
| EOA, cm² | 1.8  [1.5; 2.1] | 1.7  [1.4; 2.0] | 1.6  [1.3; 2.0] | 1.6  [1.1; 1.9] | 1.50  [1.4; 1.7] | 1.4  [1.2; 2.0] | 1.6  [1.5; ] | 1.8  [1.8; 1.8] |
| Pmean, mmHg | 8.9  [6.0; 12.0] | 9.0  [6.5; 13.0] | 9.0  [6.0; 12.0] | 9.8  [7.0; 13.8] | 8.0  [5.0; 12.0] | 7.5  [6.0; 10.5] | 7.0  [5.0; 9.8] | 17.0  [8.0; 38.0] |
| Pmax, mmHg | 16.0  [12.0;  22.0] | 16.0  [12.0;  23.0] | 16.2  [11.0;  22.8] | 17.0  [12.0;  23.5] | 16.0  [11.0;  21.0] | 15.0 [10.3;  19.1] | 16.0  [11.0;  21.8] | 36.0  [15.5;  72.0] |
| ≥moderate AR | 83 (9.0) | 38(11.0) | 14 (7.9) | 9 (10.1) | 4(10.8) | 2 (12.5) | 0 (0.0) | 0 (0.0) |
| Values are n (%), mean ± standard deviation or median [interquartile range]  AR, aortic regurgitation, EOA, Effective orifice area; LVEF, left ventricular ejection fraction; Pmean, mean transvalvular pressure gradient; Pmax, Peak transvalvular pressure gradient | | | | | | | | |

**Supplementary table 3** – Echocardiographic parameters over time of the follow-up population

|  | 1 year | 3 years | 5 years | 7 years | 9 years | 10 years | 11 years | 12 years |
| --- | --- | --- | --- | --- | --- | --- | --- | --- |
| Follow-up-population (n=56) | n=42 | n =24 | n =21 | n =16 | n =37 | n =16 | n =4 | n =5 |
| LVEF, % | 60.5  [54.0; 67.3] | 59.0  [50.0;  67.0] | 55.0  [52.0; 62.5] | 58.0  [47.0; 64.0] | 52.0  [49.0;  57.0] | 54.5  [48.3; 63.8] | 55.5  [27.8;  63.0] | 61.0  [49.0;  65.5] |
| EOA, cm² | 1.8  [1.5; 2.3] | 1.8 [  1.3; 2.2] | 1.7  [1.3; 2.5] | 1.7  [1.1; 2.1] | 1.5  [ 1.4; 1.7] | 1.4 [  1.2; 2.0] | 1.6  [1.5; ] | 1.8  [1.8; 1.8] |
| Pmean, mmHg | 9.2  [6.1; 10.8] | 7.9  [5.3; 13.5] | 8.6  [6.5; 12.5] | 9.0  [7.0; 12.0] | 8.0  [5.0; 12.0] | 7.5  [6.0;10.5] | 7.0  [5.0; 9.8] | 17.0  [8.0;38.0] |
| Pmax, mmHg | 16.0  [12.0; 20.5] | 14.3  [9.5; 25.5] | 16.6  [12.5; 23.0] | 17.3  [15.0  ;21.3] | 16.0  [11.0;  21.0] | 15.0  [10.3; 19.1] | 16.0  [11.0;  21.8] | 36.0  [15.5;  72.0] |
| ≥moderate AR | 2 (4.8) | 2(8.3) | 2(9.5) | 0 (0.0) | 4(10.8) | 2(12.5) | 0(0.0) | 0(0.0) |
| Values are n (%), mean ± standard deviation or median [interquartile range]  LVEF, left ventricular ejection fraction; EOA, effective orifice area; Pmean, mean transvalvular pressure gradient; Pmax, peak transvalvular pressure gradient AR, aortic regurgitation | | | | | | | | |

**Supplementary table 4** – Paired comparison of echocardiographic parameters for patients treated with SE THV and BE THV within the extended follow-up between timepoint of discharge and long-term follow-up

|  | THV | At discharge | At long-term follow up | p-value |
| --- | --- | --- | --- | --- |
| LVEF, % | SEV | 61.0 [54.3; 66.0] | 54.0 [4.3; 60.8] | 0.019 |
|  | BEV | 59.6 [51.0; 68.7] | 54.0 [48.5; 58.0] | 0.023 |
| EOA, cm² | SEV | 2.0 [1.7; 2.6] | 1.7 [1.4; 2.1] | 0.019 |
|  | BEV | 1.7 [1.2; 2.1] | 1.4 [1.2;1.5] | 0.260 |
| Pmean, mmHg | SEV | 7.4 [5.2; 10.9] | 6.0 [ 5.0; 10.0] | 0.673 |
|  | BEV | 8.7 [6.1; 11.4] | 10.0 [6.0; 17.0] | 0.081 |
| Pmax, mmHg | SEV | 15.5 [ 10.0; 20.4] | 12.0 [9.0; 18.8] | 0.556 |
|  | BEV | 15.9 [12.1; 21.7] | 21.0 [ 14.5; 31.0] | 0.102 |
| ≥ moderate AR | SEV | 1 (2.8%) | 3 (8.3%)  3x paravalvular | 0.157 |
|  | BEV | 1 (5.0%) | 3 (15.0%)  1x paravalvular, 2x transvalvular | 0.157 |
| Values are n (%), mean ± standard deviation or median [interquartile range]  THV, transcatheter heart valve; SEV, self-expandable valve; BEV, balloon-expandable valve, LVEF, left ventricular ejection fraction; EOA, Effective orifice area; Pmean, mean transvalvular pressure gradient; Pmax, Peak transvalvular pressure gradient AR, aortic regurgitation | | | | |

**Figures**


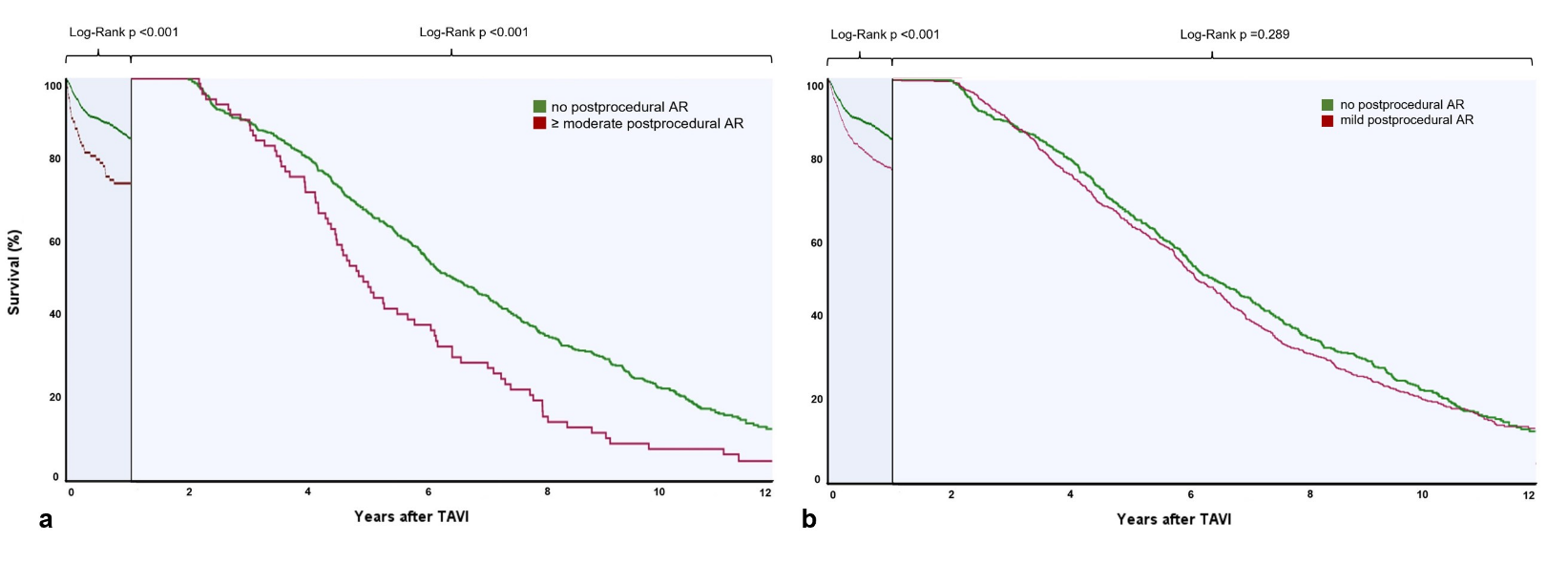
**Supplementary figure 1 -** Landmark analysis at 1 year stratified by the presence or absence of post-procedural AR

Kaplan-Meier curves before and after the landmark of 1 year of patients with no versus mild postprocedural aortic regurgitation (a) and no versus more than mild postprocedural aortic regurgitation (b).
AR=aortic regurgitation


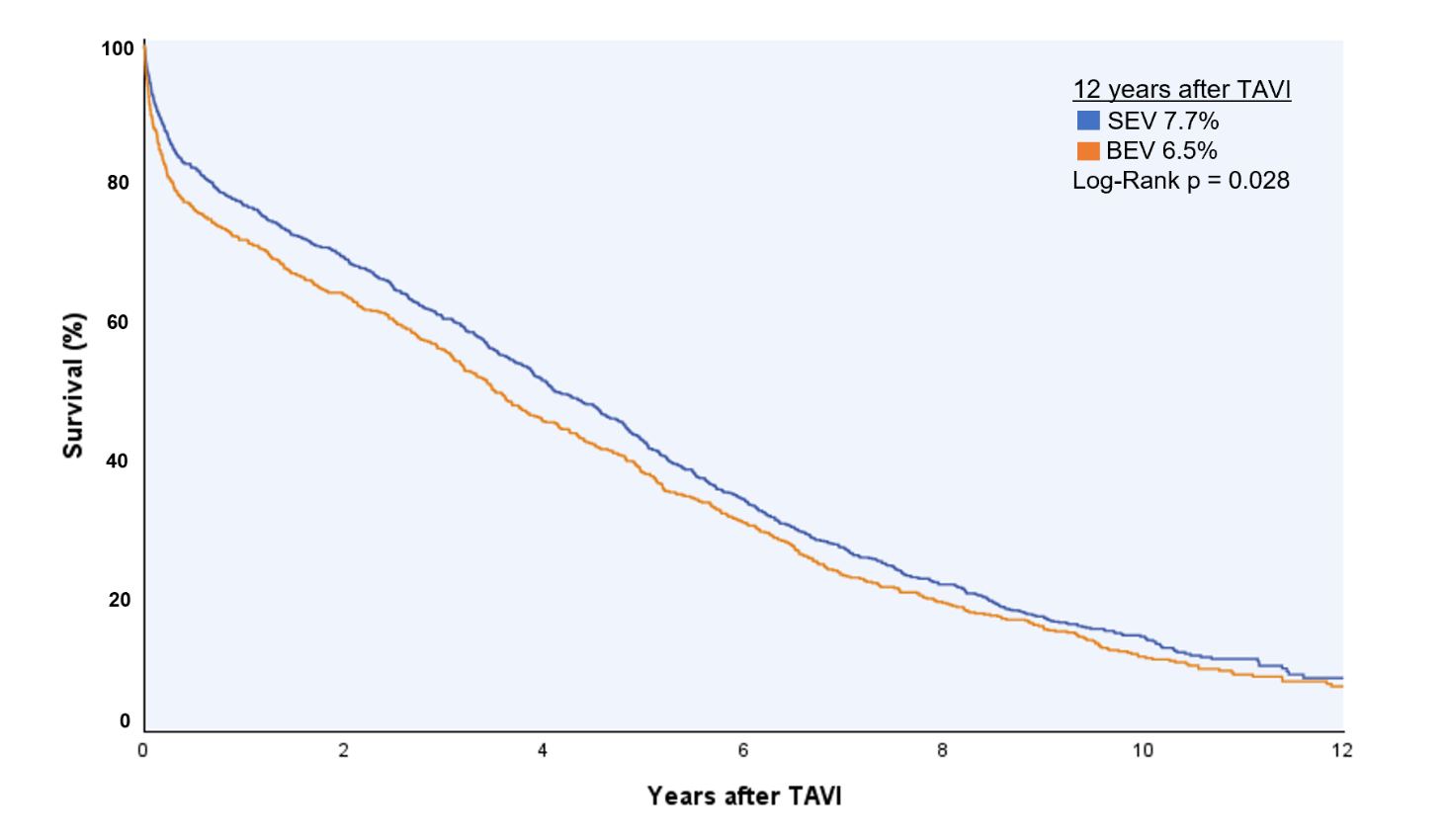
**Supplementary figure 2** – Comparison of the survival between patients with balloon-expandable and self-expanding
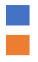
THV

| **Number at risk** | |  |  |  |  |  |
| --- | --- | --- | --- | --- | --- | --- |
| 1073 | 711 | 519 | 327 | 200 | 79 | 11 |
| 752 | 455 | 313 | 206 | 122 | 54 | 18 |
|  | | | | | | |

TAVI = Transcatheter aortic valve implantation; BE = Balloon-expandable valve; SE = Self-expandable valve
